# Mucinous histology predicts for poor response rate and overall survival of patients with colorectal cancer and treated with first-line oxaliplatin- and/or irinotecan-based chemotherapy

**DOI:** 10.1038/sj.bjc.6604955

**Published:** 2009-03-03

**Authors:** V Catalano, F Loupakis, F Graziano, U Torresi, R Bisonni, D Mari, L Fornaro, A M Baldelli, P Giordani, D Rossi, P Alessandroni, L Giustini, R R Silva, A Falcone, S D'Emidio, S L Fedeli

**Affiliations:** 1 Medical Oncology, Azienda Ospedaliera ‘Ospedale San Salvatore’, Pesaro, Italy; 2 Medical Oncology, Azienda USL 6, Istituto Toscano Tumori, Livorno, Italy; 3 Medical Oncology, Ospedale di Macerata, Macerata, Italy; 4 Medical Oncology, Ospedale ‘A Murri’, Fermo, Italy; 5 Medical Oncology, Ospedale ‘E Profili’, Fabriano, Italy; 6 Data Management, Department of OncoHematology, Azienda Ospedaliera ‘Ospedale San Salvatore’, Pesaro, Italy

**Keywords:** mucinous, colorectal cancer, chemotherapy, irinotecan, oxaliplatin, 5-fluorouracil

## Abstract

The objective of this study was to investigate the efficacy of first-line chemotherapy containing irinotecan and/or oxaliplatin in patients with advanced mucinous colorectal cancer. Prognostic factors associated with response rate and survival were identified using univariate and multivariate logistic and/or Cox proportional hazards analyses. The population included 255 patients, of whom 49 (19%) had mucinous and 206 (81%) had non-mucinous colorectal cancer. The overall response rates for mucinous and non-mucinous tumours were 18.4 (95% CI, 7.5–29.2%) and 49% (95% CI, 42.2–55.8%), respectively (*P*=0.0002). After a median follow-up of 45 months, median overall survival for the mucinous patients was 14.0 months compared with 23.4 months for the non-mucinous group (hazard ratio (HR), 1.74; CI 95%, 1.27–3.31; *P*=0.0034). After adjustment for significant features by multivariate Cox regression analysis, mucinous histology was associated with poor overall survival (HR, 1.593, 95% CI, 1.05–2.40; *P*=0.0267), together with performance status ECOG 2, number of metastatic sites ⩾2, and peritoneal metastases. This retrospective analysis shows that patients with mucinous colorectal cancer have poor responsiveness to oxaliplatin/irinotecan-based first-line combination chemotherapy and an unfavourable prognosis compared with non-mucinous colorectal cancer patients.

Colorectal cancers are derived from the cells of the colonic epithelium, and they are mainly constituted by non-mucinous cancers. However, mucinous carcinomas make up 10–20% of all colorectal cancers (Symonds and Vickery, 1976; Minsky *et al*, 1987; Green *et al*, 1993), with different clinicopathological characteristics, distinct genetic profiles, and histogenic pathways (Parham, 1923; Hanski, 1995; Zhang *et al*, 1999; Kim *et al*, 2005; Song *et al*, 2005). It has also been hypothesised that a mucinous pathway of carcinogenesis leads to a mucinous phenotype (Hanski, 1995). Mucinous adenocarcinoma is characterised by abundant extracellular mucin produced by tumour cells. By definition, a 50% or greater mucinous component is required for the designation of mucinous colorectal carcinoma (Hamilton and Aaltonen, 2000). This subtype of tumour is to be differentiated from signet ring cell carcinoma that is constituted by single tumour cells with intracytoplasmic mucin displacing their nuclei aside with 50% or more of such components. The prognostic significance of mucinous carcinoma is controversial. In some studies, mucinous histology has been shown to be an independent negative prognostic factor (Connelly *et al*, 1991; Green *et al*, 1993; Secco *et al*, 1994), but not in others (Minsky *et al*, 1987; Green *et al*, 1993; Enriquez *et al*, 1998; Consorti *et al*, 2000). Both the American Joint Committee on Cancer and the College of American Pathologists consider that the mucinous subtype has not been proven as a statistically significant prognostic factor independent of histological grade (Compton *et al*, 2000a, 2000b).

For the treatment of advanced colorectal cancer, after decades in which 5-fluorouracil (5-FU) was the only drug approved, irinotecan (IRI) and oxaliplatin (OXA) have been added during the last decade to the armamentarium of agents with activity in colorectal cancer, setting a new benchmark of survival for patients with unresectable advanced colorectal at around 20 months (Grothey *et al*, 2004; Goldberg *et al*, 2007). Interestingly, with the more recent incorporation of biological therapies, such as bevacizumab and cetuximab, overall survival (OS) of advanced colorectal cancer patients has been further improved. Now, various treatment options combining cytotoxic and targeted therapies are currently available for these patients (Grothey and Marshall, 2007).

The effect of mucinous histology on advanced colorectal cancer patients treated with first-line chemotherapy has been only recently examined by Negri *et al* (2005). In this report, 45 patients with advanced mucinous colorectal cancer have been shown to have a poorer response to 5-FU-based first-line chemotherapy and to have reduced survival compared with 90 patients with non-mucinous colorectal cancer. The overall response rate and median survival for mucinous colorectal cancer were 22% and 11.8 months compared with 47% (*P*=0.0058) and 17.9 months (*P*=0.0372) for non-mucinous tumours (Negri *et al*, 2005). No data were available on the effect of mucinous histology over the treatment efficacy of first-line regimens containing IRI and OXA. The aim of this analysis was to investigate the response rate and the OS of fluoropyrimidines in combination with IRI and/or OXA as first-line chemotherapy in patients with mucinous colorectal carcinoma.

## Patients and methods

The population consisted of 255 consecutive unselected patients who had undergone first-line chemotherapy for colorectal cancer at five oncology departments between September 2001 and December 2006. All patients had histologically confirmed diagnosis of colorectal adenocarcinoma, unidimensionally measurable disease, first-line chemotherapy containing fluoropyrimidines plus IRI and/or OXA, adjuvant/neoadjuvant treatment completed more than 6 months earlier, adequate hematological/clotting, hepatic, renal, and cardiac functions. Moreover, patients were excluded if they had received prior chemotherapy for metastatic colorectal cancer, had previous malignancy within 5 years (except for basal cell skin cancer or *in situ* carcinoma of the cervix), and were from families with familial adenomatous polyposis or hereditary non-polyposis colorectal with a highly penetrant genetic predisposition to colorectal cancer.

The pathologists from the five referral hospitals were asked to review tumour specimens and assessed the tumour type. To avoid evaluator variability in the patients, all the pathologists were not aware of the clinical results. By definition, tumours with mucinous histology had mucin constituting more than 50% of tumour volume. The colorectal adenocarcinomas without any mucinous or <50% of the mucinous component were designated as non-mucinous carcinoma (Hamilton and Aaltonen, 2000). Tumours with signet ring cells component and undifferentiated carcinoma were excluded from the analysis.

The following data were collected from the hospital records for each patient: sex, age, performance status (PS) evaluated according to the Eastern Cooperative Oncology Group (ECOG) criteria, primary tumour location, histology, earlier resection of the primary tumour, earlier tumour location, adjuvant therapy (chemotherapy and/or radiotherapy); baseline haemoglobin, CEA, and CA19-9 levels; number and sites of metastatic disease; regimen used as first-line treatment (containing fluoropyrimidines plus IRI, OXA, or both), and objective response to treatment. Patients receiving fluoropyrimidines alone or biologic agents (bevacizumab and cetuximab) as first-line chemotherapy were excluded from the analysis. Laboratory variables were initially recorded as continuous variables and later dichotomised according to the normal upper limit. Primary tumours were assigned to one of the two anatomical sites: right-sided colon (arising in the caecum, ascending colon, hepatic flexure, and transverse colon); left-sided colon (arising in the descending colon, sigmoid colon, rectosigmoid junction, and rectum).

### Treatment protocols and evaluation of response

The following first-line regimens were used to treat this population: (i) FOLFOX: OXA 85 mg m^−2^ day 1, leucovorin 200 mg m^−2^ day 1–2, bolus 5-FU 400 mg m^−2^ day 1–2, 22 h continuous infusion 5-FU 600 mg m^−2^ day 1–2, every 2 weeks; (ii) XELOX: capecitabine 1000 mg m^−2^ b.i.d. day 1–14, OXA 100–130 mg m^−2^ day 1, every 3 weeks; (iii) FOLFIRI: IRI 180 mg m^−2^ day 1, leucovorin 200 mg m^−2^ day 1–2, bolus 5-FU 400 mg m^−2^ day 1–2, 22 h continuous infusion 5-FU 600 mg m^−2^ day 1–2, every 2 weeks; (i.v.) XELIRI: capecitabine 1,000 mg m^−2^ b.i.d. day 1–14, IRI 250 mg m^−2^ i.v. day 1, every 3 weeks; (v) FOLFOXIRI: IRI 165 mg m^−2^ followed by OXA 85 mg m^−2^ leucovorin 200 mg m^−2^, and 5-FU 3200 mg m^−2^ administered as a 48-h flat continuous infusion, every 2 weeks.

Response evaluation criteria in solid tumour (RECIST) guidelines were used to define all responses (Therasse *et al*, 2000). All radiology studies were reviewed for confirming the treatment outcomes.

### Statistical analysis

The two groups of patients were compared using 2 × 2 tables for binary factors using the *χ*^2^-test, or the Fisher's exact test where appropriate. OS was calculated from the starting date of first-line chemotherapy until death of any cause, or censored at last follow-up visit. Time-to-progression (TTP) was calculated from the starting date of first-line chemotherapy to the date of progression (per investigator assessment), or death from any cause. Survival data were analysed using the Kaplan–Meier product-limit method. Comparison of survival curves was carried out using the log-rank test. The first part of the analysis consisted of the univariate comparison of survival functions for factors that could potentially affect the survival time using the log-rank test. Then, we performed a multivariate analysis using stepwise Cox proportional hazards regression modelling. *P*-values <0.05 were considered statistically significant, and all *P*-values correspond to two-sided significance tests. Approval of the study was obtained from local research and ethics committees.

## Results

The characteristics of 255 patients are presented in Table 1. Forty-nine patients (19%) had a histologically confirmed diagnosis of mucinous colorectal cancer. There were 153 male and 102 female patients, with a median age of 67 years (range, 43–89). Mucinous tumours were more frequently located into the right colon (55% compared with 29%, respectively; *P*=0.002). More patients in the mucinous group had ⩾2 metastatic sites compared with non-mucinous patients (47 and 33%, respectively; *P*=0.096). Liver, peritoneum, and lymph nodes were the most common metastatic sites in patients with mucinous cancers, whereas liver, lungs, and peritoneum were most common in patients with non-mucinous cancers. The peritoneum was more commonly noted in patients with mucinous colorectal cancer (39% compared with 12% of patients with non-mucinous tumours; *P*<0.001), whereas liver and lung metastases were found in 76 and 28% of the non-mucinous group compared with 53 and 12% of the mucinous group, respectively (*P*=0.002 and *P*=0.034, respectively). Only two patients had unresectable locally advanced disease.

### Chemotherapy regimens

Details of first-line regimens used for each patient are shown in Table 1. In all, 66% of patients were treated with OXA-based regimens, 26% of patients with IRI-based regimens, and 8% of patients with OXA/IRI-based chemotherapy. There was no significant difference between mucinous and non-mucinous groups in terms of treatment regimens. Moreover, no significant difference in the mean number of administered courses of first-line chemotherapy between mucinous and non-mucinous tumours (8 and 10 cycles, respectively) was encountered. A total of 143 (69%) patients with non-mucinous tumour (IRI-based=94 patients; OXA-based=33 patients; IRI/OXA-based=7 patients; and =9 patients) and 31 (63%) patients with mucinous tumours (IRI-based=22 patients; OXA-based=5 patients; IRI/OXA-based=2 patients; and other=2 patients) received second-line chemotherapy.

### Treatment response

All the patients had measurable disease and were evaluated for response (Table 2A and B). Ten patients discontinued the treatment prematurately because of early disease progression, seven (3%) patients with non-mucinous, and three (6%) with mucinous cancer (*P*=NS). Fourteen patients achieved complete response and 96 patients achieved partial remission,; thus, the overall response rate was 43.1% (95% CI, 37.1–49.2). In the mucinous colorectal cancer group, nine patients reported a partial response for an overall response rate of 18.4% (95% CI, 7.5–29.2%). In the non-mucinous group, 14 patients achieved complete response and 87 achieved partial remission for an overall response rate of 49.0% (95% CI, 42.2–55.8%). The difference of response rate between the two groups was statistically significant (*P*=0.0002). On multivariate analysis (Table 3), patients with PS 0–1 (risk ratio, 6.06; 95% CI, 1.32–27.7; *P*=0.02), non-mucinous histology (risk ratio, 3.41; 95% CI, 1.53–7.61; *P*=0.002), and without peritoneal metastases (risk ratio, 2.70; 95% CI, 1.14–6.37; *P*=0.026) had a significantly increased probability of tumour response to chemotherapy.

### Survival

**Figure 1:**
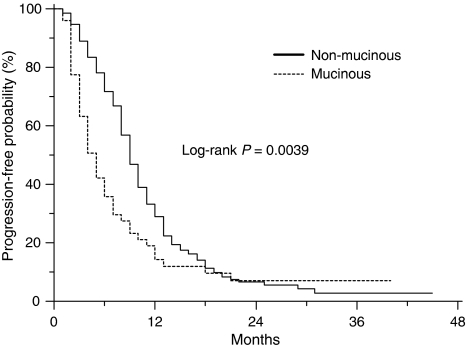
Time-to-progression for patients with mucinous and non-mucinous colorectal cancer (*n*=255).

**Figure 2:**
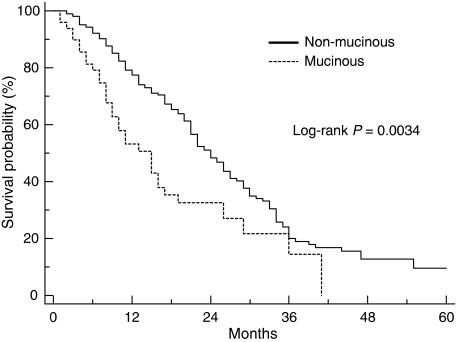
Overall survival for patients with mucinous and non-mucinous colorectal cancer (*n*=255).

After a median follow-up of 45 months, the median TTP for mucinous colorectal cancer patients was 4.1 months compared with 8.6 months for the non-mucinous group (*P*=0.0039) (Figure 1). The HR for risk of progression for patients with mucinous colorectal cancer compared with non-mucinous tumours was 1.57 (95% CI, 1.21–2.74). The median OS for the mucinous colorectal cancer patients was 14.0 months compared with 23.4 months in the non-mucinous colorectal cancer group (HR=1.74; 95% CI, 1.27–3.31; *P*=0.0034; Figure 2). The 1-year OS was 53.1% (95% CI, 45.5–60.7%) for the mucinous group compared with 77.4% (95% CI, 74.4–80.4%) for the non-mucinous group.

The univariate analysis (Table 4) showed the other four variables to be significantly associated with poor survival: PS ECOG 2, number of metastatic sites ⩾2, peritoneal metastasis, and haemoglobin ⩽12 g l^−1^. Two variables, CEA and CA19-9, had missing data, and they were not included in the multivariate analysis. After correcting for significant prognostic factors by multivariate Cox regression analysis, mucinous histology was confirmed as poor prognostic factor (HR, 1.593; 95% CI, 1.05–2.40; *P*=0.0267; Table 5). Multivariate regression analysis (Table 5) also found PS ECOG 2, number of metastatic sites ⩾2, and peritoneal metastases to be negative independent prognostic factors.

## Discussion

It is generally recognised that mucinous tumours of the colon and rectum have a worse prognosis than non-mucinous tumours (Umpleby *et al*, 1985; Green *et al*, 1993; Consorti *et al*, 2000), and they occur more frequently in the proximal colon (Umpleby *et al*, 1985; Green *et al*, 1993; Sarli *et al*, 2008), metastasise with high frequency to lymph nodes and to the peritoneum (Umpleby *et al*, 1985; Minsky *et al*, 1987), are more prone to local recurrence (Umpleby *et al*, 1985), and are typically diagnosed at an advanced stage (Green *et al*, 1993; Younes *et al*, 1993).

Published literature presents conflicting results on the association between worse prognosis and mucinous histology of colorectal cancer. However, the interpretation of results may be arduous for many reasons. Data are mostly derived from series that assessed the prognosis of patients treated with curative surgery alone, at different stages of disease, and did not evaluate closely a role for chemotherapy in this subset of patients (Green *et al*, 1993; Younes *et al*, 1993; Consorti *et al*, 2000; Kanemitsu *et al*, 2003). Moreover, the geographical variations in the epidemiology of mucinous colorectal cancer may likely account for the conflicting results (Sarli *et al*, 2008).

Our analysis showed a highly statistically significant poor survival for patients with mucinous tumours compared those with non-mucinous tumours (14 months *vs* 23.4 months, respectively). All the patients included in the present analysis had advanced colorectal cancer and were treated with first-line chemotherapy containing IRI and/or OXA in addition to fluoropyrimidines, considered as standard drugs for this disease at that time. Characteristics of patients were well balanced according to the different clinicopathological variables, except for a higher proportion of patients with mucinous colorectal cancer who had peritoneal metastases and were right-sided. Conversely, more patients with non-mucinous tumours had liver and lung metastases. These findings were also found in earlier studies (Umpleby *et al*, 1985; Minsky *et al*, 1987; Negri *et al*, 2005), and, to our opinion, these imbalances cannot justify the poor prognosis of patients with mucinous carcinomas. Moreover, the multivariate analysis confirmed the independent poor prognostic role of mucinous histology (HR 1.593, 95% CI 1.05–2.40; *P*=0.0267), together with PS, number of metastatic sites, and peritoneal metastasis.

In advanced colorectal cancer, the less responsiveness to first-line chemotherapy of mucinous tumours compared with non-mucinous tumours has been only recently reported in a case–control study by the group of the Royal Marsden Hospital (Negri *et al*, 2005). Our aim was to confirm the same results in a similar subset of patients, but receiving IRI and OXA in addition to fluoropyrimidines as first-line chemotherapy. Mucinous colorectal cancers had a response rate to IRI and/or OXA-based chemotherapy of 18.4% compared with 49% for non-mucinous tumours (*P*=0.0002). Logistic regression analysis revealed that histology, namely, mucinous, together with bad PS, and peritoneal metastasis were independent predictive factors for poor response.

The mechanisms that lead to this significant difference in IRI, OXA, and fluoropyrimidine sensitivity of mucinous tumours compared with non-mucinous tumours are unknown. Mucinous tumours have been characterised by a number of genetic and biological features, which may explain in part the different behaviour compared with that of non-mucinous tumours. Mucinous colorectal carcinomas have higher incidence of high degree of microsatellite instability (MSI-H) (Kakar *et al*, 2004; Song *et al*, 2005; Ogino *et al*, 2006; Tanaka *et al*, 2006; Sarli *et al*, 2008), K-ras mutation (Zhang *et al*, 1999; Bazan *et al*, 2002; Ogino *et al*, 2006), BRAF mutation (Song *et al*, 2005; Li *et al*, 2006; Ogino *et al*, 2006; Tanaka *et al*, 2006), and less expression of p53 (Zhang *et al*, 1999; Ogino *et al*, 2006) than do non-mucinous colorectal cancer. Given the unclear significance and heterogeneity of mucinous colorectal cancers, it seems difficult to find possible explanations for the relatively chemoresistance of such tumours. It is recognised that defective DNA mismatch repair (MMR) leads to MSI and results in resistance to many antineoplastic drugs, such as antimetabolites, alkylating, and platinum agents, and inhibitors of topoisomerases. There are some evidences *in vitro* that suggest a correlation between response to 5-FU, OXA, and IRI and MSI (Magrini *et al*, 2002; Arnold *et al*, 2003; Warusavitarne and Schnitzler, 2007). Our analysis plan did not include an MSI analysis. First, we focused on the clinical role of mucinous histology without performing molecular analysis, as the simple knowledge on the histopathological mucinous feature may be *per se* a relevant information. Second, two recently published studies (Braun *et al*, 2008; Müller *et al*, 2008) have shown low MSI in colorectal carcinomas (about 4%), and no significant association with treatment (OXA- and IRI-based) outcomes in terms of response rate and OS; therefore, the use of this marker may be of limited value. The discrepancy between such a low percentage of MSI and the higher percentage of MSI reported in earlier studies might be the result of many reasons, such as a variable definition of MSI-H, the use of different markers, or a consequence of the selection of patients (Popat *et al*, 2005; Müller *et al*, 2008).

Recently, Glasgow *et al* (2005) analysed some molecular markers for response to chemotherapy in mucinous and non-mucinous Dukes C colorectal cancer. The authors found an overexpression of TS and GSTP1 (glutathione *S*-transferase pi) genes in mucinous tumours. As GSTP1 is a major rout of detoxification of platinum agents, one could expect that the overexpression of TS and GSTP1 genes in mucinous tumours may be responsible for decreased clinical response to treatment with 5-FU and OXA.

Several markers, oncogenes and suppressor genes, multidrug-resistance-related proteins, and genomic polymorphisms that influence DNA metabolism, DNA damage, programmed cell death, and angiogenesis may be responsible of colorectal cancer patient's variation in response to chemotherapy. It is important to stress that before clinical application, any biomarker need to be independently validated. Moreover, for mucinous carcinomas, many of the above reported biomarkers may differently be expressed in each tumour, and this could make very difficult to potentially predict response to chemotherapy for this heterogeneous tumours.

Another key point of discussion is the integration of conventional cytotoxic agents with novel biologic agents. Targeted agents enhance the efficacy of conventional cytotoxic agents (Goldberg *et al*, 2007). We actually lack data investigating the outcome of patients with mucinous colorectal cancer treated with novel agents and traditional drugs (fluoropyrimidines, IRI, and OXA).

Despite the possible limitations of this retrospective analysis, the observations of this study are consistent with those of the group of the Royal Marsden Hospital (Negri *et al*, 2005), which showed that mucinous advanced colorectal cancer patients treated with first-line fluoropyirimidine-based chemotherapy have similar response rate and OS. However, these retrospective analyses were based on a rather small number of patients. It would be very worthwhile to try to confirm these findings in an analysis of large, prospective, randomised trials, such as N9741, NO16966, and so on. The informations derived on mucinous histology may help researchers and practitioners in designing future studies (e.g., stratification of patients according to the mucinous histology) and in making clinical decisions that will improve the outcome of patients with such histological type. Trying to identify subsets of patients who are likely to derive more benefit from a particular treatment not only helps to derive greater efficacy, but also spares many patients from unnecessary toxicity.

In conclusion, this retrospective analysis on advanced colorectal cancer showed poor responsiveness and prognosis for patients with mucinous colorectal cancer treated with first-line chemotherapy containing 5-FU, IRI, and OXA. Further investigations should be carried out to better characterise the genetic profile and the pharmacological markers, which could explain the unfavourable responsiveness and prognosis of mucinous colorectal tumours. Assessment of clinical outcome of mucinous colorectal cancer treated with cytotoxic drugs and novel agents is highly warranted.

## Figures and Tables

**Table 1:** Patient characteristics

**Characteristic**	**Non-mucinous (*n*=206)**	**Mucinous (*n*=49)**	***P*-value**	**Overall (*n*=255)**
*Sex*				
Male	122 (59%)	31 (63%)	0.721	153 (60%)
Female	84 (41%)	18 (37%)		102 (40%)
Median age, years (range)	67 (43–84)	67 (45–89)	0.927	67 (43–89)
				
*Peformance status (ECOG)*				
0	102 (50%)	21 (43%)	0.263	123 (48%)
1	91 (44%)	22 (45%)		113 (44%)
2	13 (6%)	6 (12%)		19 (8%)
				
*Primary tumour site*				
Right-sided	61 (29%)	27 (55%)	0.002	88 (34%)
Left-sided	144 (70%)	22 (45%)		16 (65%)
Synchronous primaries	1 (1%)	0 (0%)		1 (1%)
				
*Grading*				
1	22 (11%)	3 (6%)	0.583	25 (10%)
2	121 (59%)	26 (53%)		147 (57%)
3	52 (25%)	14 (29%)		66 (26%)
Missing	11 (5%)	6 (12%)		17 (7%)
Resection of primary tumour	144 (70%)	31 (63%)	0.466	175 (69%)
Earlier adjuvant chemotherapy	74 (36%)	19 (39%)	0.835	93 (36%)
				
*Number of metastatic sites*				
0–1	138 (67%)	26 (53%)	0.096	164 (64%)
⩾2	68 (33%)	23 (47%)		91 (36%)
				
*Site of metastatic disease*				
Liver	157 (76%)	26 (53%)	0.002	183 (72%)
Peritoneum	24 (12%)	19 (39%)	<0.001	43 (17%)
Lymph node	18 (9%)	8 (16%)	0.188	26 (10%)
Lung	58 (28%)	6 (12%)	0.034	64 (25%)
CNS	4 (2%)	0 (%)		4 (2%)
Bone	3 (1%)	1 (2%)		4 (2%)
Other	20 (10%)	15 (30%)		35 (14%)
Haemoglobin level, g l^−1^	12.9 (8.2–17.4)	12.2 (8.2–16.1)	0.077	12.7 (8.2–17.4)
CEA, ng ml^−1^	18.4 (0–1084)	14.4 (0.9–1151)	0.851	17.0 (0–1151)
CA19-9, U ml^−1^	456 (0–20000)	122 (3.2–48000)	0.928	45.0 (0–48000)
				
*First-line regimen*				
IRI-based regimen	56 (27%)	9 (18%)	0.730	65 (26%)
OXA-based regimen	135 (66%)	34 (70%)	0.275	169 (66%)
IRI/OXA-based regimen	15 (7%)	6 (12%)		21 (8%)

CEA=carcinoembryonic antigen; CNS=central nervous system; ECOG=Eastern Cooperative Oncology Group; IRI=irinotecan; OXA=oxaliplatin.

**Table 2:** Response rate according to histology (A) and regimen of first-line chemotherapy (B)

*(2A)*			
**Response**		**Mucinous (*n*=49)**	**Non-mucinous (*n*=206)**
Complete response		0	14 (6.8%)
Partial response		9 (18.4%)	87 (42.2%)
Overall response rate, % (95% CI)		18.4 (7.5–29.2)	49.0 (42.2–55.8)
Stable disease		18 (36.7%)	57 (27.7%)
Progressive disease		22 (44.9%)	48 (23.3%)
			
*(2B)*			
**Chemotherapy regimen**	***N***	**Mucinous responders** a	**Non-mucinous responders** a
OXA-based	169	6/34 (17.6%)	57/135 (42.2%)
IRI-based	65	1/9 (11.1%)	32/56 (57.1%)
OXA/IRI-based	21	2/6 (33.3%)	12/15 (80.0%)

CI=confidence interval; IRI=iriotecan; OXA=oxaliplatin.

a Responders=complete plus partial responses according to the RECIST.

**Table 3:** Multivariate logistic regression model for tumour response to 1st line chemotherapy (*n*=255)

**Variable**	**Risk ratio (95% CI)**	***P*-value**
*Performance status (ECOG)*		
0–1	6.06 (1.32–27.7)	0.020
		
*Histology*		
Nonmucinous	3.41 (1.53–7.61)	0.002
No peritoneal metastases	2.70 (1.14–6.37)	0.026

ECOG=Eastern Cooperative Oncology Group; CI=confidence interval.

**Table 4:** Factors associated with overall survival in the univariate analysis

**Variable**	***n***	**MST (months)**	**1-year-survival (%)**	***P*-value**
*Sex*				
Male	153	21.2	74.3	
Female	102	22.6	70.9	0.4040
				
*Age*				
⩽65 years	111	21.7	76.9	
>65 years	144	21.5	69.8	0.0828
				
*Performance status (ECOG)*				
0–1	236	23.0	76.9	
2	19	6.2	22.6	<0.0001
				
*Primary tumor site*				
Right-sided	88	21.0	75.6	
Left-sided	166	21.5	71.6	0.5222
				
*Histology*				
Nonmucinous	206	23.4	77.4	
Mucinous	49	14.0	53.1	0.0034
				
*Previous adjuvant treatment*				
Yes	93	27	75,3	
No	162	20,7	71,6	0.0619
				
*Number of metastatic sites*				
0–1	164	25.2	80.0	
⩾2	91	14.2	59.9	0.0003
				
*Liver metastasis*				
Yes	183	21.8	75.6	
No	72	20.5	66.4	0.3537
				
*Peritoneal metastasis*				
Yes	43	11.3	47.9	
No	212	23.7	78.0	<0.0001
				
*Nodal metastasis*				
Yes	26	14.6	61.7	
No	229	22.6	74.1	0.0581
				
*Lung metastasis*				
Yes	64	26.0	83.6	
No	191	20.6	69.5	0.190
				
*Haemoglobin level*				
⩽12 g l^−1^	90	18.0	61.2	
>12 g l^−1^	165	22.4	79.0	0.0489
				
*CEA*				
⩽5 ng ml^−1^	79	24.0	79.8	
>5 ng ml^−1^	153	20.8	69.7	0.0214
				
*CA19-9*				
⩽30 U ml^−1^	117	26.3	80.9	
>30 U ml^−1^	114	17.2	65.2	0.0004

ECOG=Eastern Cooperative Oncology Group; CEA=carcinoembryonic antigen; MST=median survival time.

**Table 5:** Factors associated with a poor overall survival in mutivariate analysis (*n*=255)

**Variable**	**Hazard ratio (95% CI)**	***P*-value**
*Performance status (ECOG)*		
2	3.526 (2.07–5.99)	<0.0001
		
*Histology*		
Mucinous	1.593 (1.05–2.40)	0.0267
		
*Number of metastatic sites*		
⩾2	1.472 (1.04–2.08)	0.0300
		
*Peritoneal metastasis*		
Yes	1.588 (1.01–2.49)	0.0461
		
*Haemoglobin level*		
⩽12 g l^−1^	1.123 (0.81–1.55)	0.4870

ECOG=Eastern Cooperative Oncology Group; CI=confidence interval.
